# The Role of cAMP-PKA Pathway in Lactate-Induced Intramuscular Triglyceride Accumulation and Mitochondria Content Increase in Mice

**DOI:** 10.3389/fphys.2021.709135

**Published:** 2021-09-13

**Authors:** Siyu Chen, Lei Zhou, Jingquan Sun, Yaqian Qu, Min Chen

**Affiliations:** ^1^Institute of Sports Science, Sichuan University, Chengdu, China; ^2^School of Physical Education and Sports, Sichuan University, Chengdu, China

**Keywords:** lactate, cAMP, intramuscular triglyceride, mitochondria content, skeletal muscle

## Abstract

The glycolytic product of exercise, lactate, has long been recognized to promote lipid accumulation by activation of G-protein-coupled receptor 81 (GPR81) and inhibition of the cyclic adenosine monophosphate-protein kinase A (cAMP –PKA) pathway in adipose tissue. Whether lactate causes a similar process in skeletal muscle is unclear. Lactate might also improve mitochondria content in skeletal muscle; however, the mechanism is not clarified either. In this study, using intramuscular injection of lactate to the gastrocnemius and intraperitoneal injection of forskolin (activator of cAMP-PKA pathway), we identified the role of the cAMP-PKA pathway in lactate-induced intramuscular triglyceride accumulation and mitochondrial content increase. The intramuscular triglyceride level in the gastrocnemius increased after 5weeks of lactate injection (*p*<0.05), and this effect was blocked by forskolin injection (*p*<0.05). Corresponding expression level changes of GPR81, P-PKA/PKA, P-CREB/cAMP-response element binding protein (CREB), and proteins related to lipid metabolism suggest that lactate could induce intramuscular triglyceride accumulation partly through the inhibition of the cAMP-PKA pathway. Meanwhile, the intramuscular expression of citrate synthase (CS) and the activity of CS increased after 5weeks of lactate injection (*p*<0.05), but the change of CS expression was not blocked by forskolin injection, suggesting other mechanisms might exist. Consequently, exploration for other potential mechanisms that might contribute to the lactate-induced mitochondria content increase was conducted. We found an increase in the contents of lactate-related metabolites in skeletal muscle mitochondria after acute lactate injection (the *p-value* of each analysis is less than 0.05). LHDA was also validated to exist in mitochondria in this study. These results provide a possibility for metabolism-related mechanisms of lactate-induced mitochondria content increase. Future study is needed to validate this hypothesis. In conclusion, lactate-induced intramuscular triglyceride accumulation is achieved by inhibition of lipolysis, and this process is regulated by the cAMP-PKA pathway. Promoted lipogenesis also contributes to lactate-induced triglyceride accumulation, and this process might also be regulated by the cAMP-PKA pathway. Lactate injection might increase mitochondria content and cAMP-PKA pathway might have a limited contribution, while other metabolism-related mechanisms might play a prominent role.

## Introduction

Lactate has long been recognized to promote lipid accumulation in adipocytes. Infusion of lactate has been reported to suppress lipolysis in adipose tissue (Gold et al., 1963; Bjorntorp, 1965; Houghton et al., 1971; Boyd et al., 1974; De Pergola et al., 1989; Cai et al., 2008). This effect of lactate is known to be correlated with the activation of its receptor G-protein-coupled receptor 81 (GPR81) and the inhibition of its downstream cAMP-PKA pathway in adipose tissue (Sakurai et al., 2014). Our recent research showed that lactate could directly induce similar lipid accumulation in skeletal muscle as it does in adipose tissue (Zhou et al., 2021b). However, the mechanism is unclear.

Previous studies have validated that intramuscular triglyceride accumulation is usually accompanied by mitochondrial adaption to breakdown triglycerides more efficiently, and a positive association has also been found between intramuscular triglyceride accumulation and the expression levels of mitochondrial biogenesis proteins (Koves et al., 2013). Growing evidence suggests that lactate might act as a signaling molecule and contribute to mitochondrial adaptation in skeletal muscle (Takahashi et al., 2020). Our recent research also verified that chronic lactate injection could not only induce the intramuscular triglyceride accumulation but also increase the expression levels of mitochondria protein, suggesting increased mitochondria content after lactate administration (Zhou et al., 2021b). However, the mechanism of this lactate-induced mitochondria content increase in skeletal muscle is rarely investigated.

It is reported that mitochondria content might be modulated by the cAMP-PKA pathway. An experiment *in vitro* indicated that activation of the cAMP-PKA pathway increased the copy number of mitochondrial DNA (Bogacka et al., 2005). De Rasmo et al. (2012, 2015) also demonstrated that the cAMP-PKA pathway could regulate the expression levels of the subunits of the electron transport chain complex in mitochondria. However, in our previous study, lactate injection suppressed cAMP-PKA pathway and upregulated the expression level of PGC1-α and CS, which seems to be paradoxical. In this study, the expression level of CS and its activation were also upregulated by lactate, and increased expression of CS was not inhibited by forskolin. Hence, we presume that mechanisms other than cAMP-PKA pathway might play a prominent role in lactate-induced mitochondria content increase.

As indicated above, the mechanism whereby lactate regulates intramuscular lipid accumulation and mitochondria content is presently unclear. Here, using intramuscular injection of lactate to the gastrocnemius and intraperitoneal injection of forskolin (activator of cAMP-PKA pathway), we investigated the role of the cAMP-PKA pathway in lactate-induced intramuscular lipid accumulation and mitochondria content increase. Other possible mechanisms of increased mitochondria content after lactate injection were also explored from the aspect of energy metabolism.

## Materials and Methods

### Animals

Experiments were performed with c57bl/6 mice (7weeks of age, obtained from Chengdu DaShuo Biological Technology Co., Ltd., China). Mice were maintained on a standard rodent chow diet and water *ad libitum* under 12-h light and dark cycles. All the administration was implemented after an acclimation period of 1week.

In the first experiment aiming to clarify the time dependence of acute lactate and forskolin injection-induced variation of cAMP-PKA pathway, c57BL/6 mice were randomly assigned into 10 groups: sacrificed after acute phosphate-buffered saline injection (AP; *n*=6); sacrificed after acute DMSO injection (AD; *n*=6); sacrificed at 15, 30, 60, and 120min after acute lactate injection (AL-15, AL-30, AL-60, and AL-120; *n*=6); sacrificed at 15, 30, 60, and 120min after acute forskolin injection (AF-15, AF-30, AF-60, and AF-120; *n*=6); sacrificed at 60min after acute lactate and forskolin injection (60min interval, ALF; *n*=6).

In the subsequent chronic experiment, the animals were assigned randomly into five groups: chronic phosphate-buffered saline treated group (CP; *n*=8); chronic lactate treated group (CL; *n*=8); chronic forskolin treated group (*CF*; *n*=8); chronic DMSO treated group (CD; *n*=8); and chronic lactate and forskolin treated group (CLF; *n*=8). Each chronic group was administrated for 5weeks and sacrificed by cervical dislocation at 72h after the last injection. No death occurred during the experiment.

The procedures for the care and use of animals were approved by the Ethics Committee of Sichuan University and conform with all the applicable institutional and governmental regulations on the ethical use of animals.

### Treatment Protocol

Lactate treatment was performed with sodium lactate (C43922, Acros) solution prepared in PBS (C0221A, Beyotime). Local delivery of sodium lactate solution was performed by intramuscular injection in the gastrocnemius of the left limb (0.64ml/kg). According to a previous study (Nikooie and Samaneh, 2016), this value of lactate (0.25M) was selected to elevate lactate levels in the gastrocnemius muscle to an average value of 25mmol/L. PBS treatment was performed with PBS solution at the volume of the lactate solution and by the same injection method of the lactate treatment. Forskolin treatment was performed with 5% forskolin (F3917, SigmaAldrich) solution prepared in 4% DMSO. The delivery of forskolin solution was performed by intraperitoneal injection (5mg/kg). DMSO treatment was performed with 4% DMSO solution at the volume of the forskolin solution and by the same injection method of the forskolin treatment. Lactate and forskolin treatment was performed with lactate solution and then forskolin solution following the same injection protocols mentioned above. The time interval between the two injections is 15min.

Injections in chronic treatment groups were implemented five times a week (one injection daily for consecutive 2days, after that comes a day off; and then one injection daily for consecutive 3days, after that comes a day off) for 5weeks, and the injection in the first experiment was carried out with the same procedure of every single injection in the chronic groups.

### Determination of Blood Lactate Concentration

Portable electrochemical devices Lactate Scout and Sensors (SensLab EKF, Germany) were used to determine the blood lactate level after acute lactate injection. A previous study has proved the accuracy of this device (Romanov et al., 2020). Blood drop was excised from the aseptically treated wound of mice’s tail. After resting for 20min, resting blood lactate concentration was determined. Then mice were injected with lactate and then placed in the chamber to avoid external stimuli for the subsequent tests.

### Oil Red O Staining of Gastrocnemius

To determine the morphological effects of lactate on intramuscular lipid droplets, oil red O staining was performed using frozen tissue sections as previously described. Briefly, muscle tissue was washed with PBS, treated with 4% paraformaldehyde, and incubated in 60% oil Red O for 10min. After washing residual oil red O from muscle tissue, images were collected using an inverted microscope (NIKON Eclipse Ci).

### Immunofluorescence of Gastrocnemius

According to a previous study (Elustondo et al., 2013), the muscle of mice was fixed in 4% paraformaldehyde for 1h, treated with 100mM glycine for 30min, and permeabilized with Tris-buffered saline containing 0.1% Tween 20 (w/v) for 20min. The samples were blocked for 1h with PBS containing 1% gelatin, incubated with anti-LDHA (Proteintech, 19987-7-AP) for 1h at room temperature, washed four times with PBS, and then incubated with the secondary antibody goat anti-rabbit conjugated with Dylight 549 for another hour (711-505-152, Jackson ImmunoResearch Laboratories). Following washes, the same samples were incubated with anti-HSP60 (Invitrogen, MA3-012) for 1h at room temperature, washed four times with PBS, and then incubated with the secondary antibody goat anti-rabbit conjugated with Dylight 549 for another hour, and then washed and mounted with DAKO mounting media. Images were collected using a fluorescent confocal microscope (NIKON Eclipse Ti). Experiments were repeated three times in triplicate.

### Triglyceride Assay of Gastrocnemius

Intracellular triglycerides were assayed using a triglyceride assay kit (GPO-POD; Applygen Technologies Inc., Beijing, China). About 25±5mg of the gastrocnemius muscle of six mice of each group were weighed and lysed on ice. After 70°C heating for 10min, each sample was placed in a 96-well plate with two duplicates and then mixed with the kit’s A+B solution. After a 15min incubation at 37°C and cooling to room temperature, the resultant purple color is measured using a spectrometer at 492nm. Then the final values are normalized by each sample’s protein concentration measured by the BCA assay.

### Citrate Synthase Activity Assay of Gastrocnemius

Citrate synthase (CS) activity was assayed using a citrate synthase kit (Beijing Solarbio Science & Technology Co., Ltd., China). About 25±5mg of the gastrocnemius muscle of eight mice of each group were weighed and lysed on ice and then analyzed according to the protocol provided by the kit. The final values are normalized by each sample’s protein concentration measured by the BCA assay.

### Lactate Concentration Assay of Gastrocnemius

Lactate concentration of gastrocnemius after acute lactate injection was assayed using a lactate acid assay kit (Beijing Solarbio Science & Technology Co., Ltd., China). About 35±5mg of the gastrocnemius muscle of four mice of each group were weighed and lysed on ice and then analyzed according to the protocol provided by the kit. The final values are normalized to each sample’s fresh weight.

### Liquid Chromatography-Mass Spectrometry of Gastrocnemius

At 5min after acute injection of lactate and PBS, mice were sacrificed and mitochondria were obtained using the Mitochondria Isolation Kit (Cat: KGA827, KeyGEN, Nanjing, China): (1) Left gastrocnemius muscle tissue was collected and washed with PBS. The tissue was minced and placed in a glass homogenizer, to which was added the pre-cooled Lysis Buffer (6-fold volumes of the tissue), and then dounced 20 times with the glass homogenizer in an ice bath; (2) Homogenate was transferred into microcentrifuge tubes containing 0.2ml Medium Buffer, mixed gently and then centrifuged for 5min at 1,200×*g* and 4°C and the supernatant was collected. The supernatant of the first centrifuge was centrifuged once more and the supernatant was collected; and (3) The supernatant was transferred to a clean microcentrifuge tube. The supernatant was centrifuged at 7,000×*g* for 10min, and the supernatant was discarded. The precipitates were mitochondria.

Mixed with MeOH-H2O (8:2), mitochondria was stored at −20°C for 20min and ultrasonicated in an ice bath for 15min. The viscous mixture was centrifuged at 13,300rpm for 15min at 4°C. Then the centrifuged supernatant was collected and concentrated under high vacuum for 2h. After stored at −80°C for 48h, the concentration was redissolved in MeOH-H2O (8:2) with 13C1-Lactate. The solution was ultrasonicated in an ice bath for 10min and centrifuged at 13,300rpm for 10min at 4°C. The centrifuged supernatant was collected for mass spectrometry (ms).

Contents of L-lactic acid, succinic acid, L-malic acid, and oxalacetic acid were analyzed on an AB SCIEX QTRAP 5500 triple quadrupole mass spectrometer (AB SCIEX, Framingham, United States) equipped with a Shimadzu LC-30AD HPLC system (Shimadzu, Kyoto, Japan). About 1 μl of the mitochondria extract was injected onto a Waters Acquity UPLC HSS-T3 columna held at 30°C for chromatographic separation. The mobile phase A was water with 0.1% formic acid and mobile phase B was methanol, and gradient elution was performed. The flow rate was 0.3ml/min. All the samples were kept at 4°C during analysis.

The mass spectrometer was operated in negative ion mode, with the ion-spray voltage set at −4,000V, curtain gas at 35 (a.u.), source gas 1 and gas 2 at 50 and 40 (a.u.), respectively, and source temperature at 650°C. Data was acquired and processed using Multiquant software. Peak areas of individual metabolites were normalized against the peak areas of 13C1-Lactate and then normalized against the total protein concentration of each sample measured by the BCA assay.

### Western Blot

After the animals were sacrificed, the injected gastrocnemius was quickly excised on ice. The gastrocnemius muscle tissue was stored at −80°C and the protein in whole gastrocnemius muscle tissue was extracted later using the following protocol: approximately 100mg of the whole gastrocnemius muscle of each group (*n*=10) was homogenized in ice-cold RIPA buffer and then centrifuged at 12,000RPM for 30min at 4°C. The supernatant’s protein concentration was determined by BCA assay (Thermo): supernatant and gradient-diluted protein standards were placed in a 96-well plate with two duplicates and then mixed with the kit’s A+B solution. After a 30min incubation at 37°C and cooling to room temperature, the resultant purple color is measured using a spectrometer at 562nm. In this way, the standard curve was determined, and the protein concentration could be calculated by application of the standard curve. The supernatant was finally trimmed by PBS for western blotting analysis.

About 40–50μg protein for each lane was separated on a 10/12% SDS–PAGE gel and transferred onto a PVDF membrane. Then, blocked with 5% skimmed milk for 30–60min. Antibodies used for western blotting were Anti-Rabbit Secondary Antibody (31,460, Invitrogen), Anti-Mouse Secondary Antibody (31,460, S0002), GAPDH (AF7021, Affinity), GPR81 (PA5-75664, Invitrogen), P-CREB (AF3189, Affinity), P-CREB (AB32096, Abcam), CREB (AF6188, Affinity), CREB (AB32515, Abcam), sterol regulatory element-binding protein 1c (SREBP-1C; AF4728, Affinity), PPAR-γ (AF6284, Affinity), P-HSL (AF2206, Beyotime), CS (DF13222, Affinity), P-ACC (Abcam, ab68191), acetyl-CoA carboxylase (ACC; CST, 3676s), hormone-sensitive lipase (HSL; AF6403, Affinity), PKA (AF7746, Affinity), P-PKA (AF7246, Affinity), adipose triglyceride lipase (ATGL; AB207799, Abcam), P-ATGL (AB135093, Abcam), FASN (CST, 31,805), and β-tubulin (AB179513, Abcam). Blots were developed using Western Lightning ECL (Affinity). All the bands were analyzed with Image J. GAPDH and β-tubulin were used for the normalization of each protein to ensure the loading of equal quantities of protein.

### Statistical Analysis

Data is presented as mean±SD. The comparison between the means of two groups was assessed by the independent-samples *t*-test. The comparison among the multiple groups was assessed by one-way ANOVA. We assigned * for values below 0.05. Statistical graphs and statistical analyses were carried out using Prism 8 (GraphPad).

## Results

### The Effect of Acute Intramuscular Lactate Injection on the Injected Gastrocnemius Lactate Concentration

**Figure 1:**
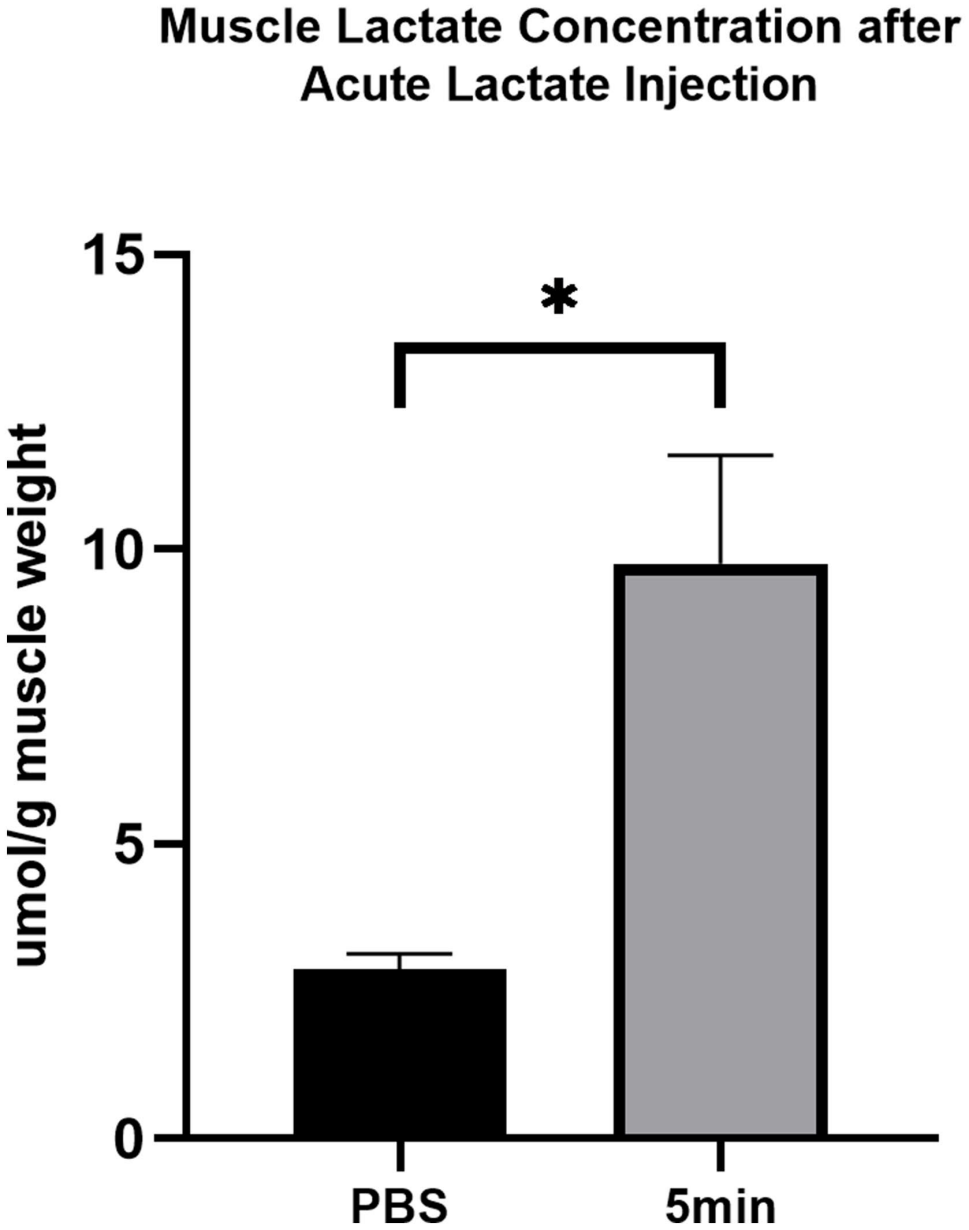
The effect of acute intramuscular lactate injection on the injected gastrocnemius lactate concentration. The muscle lactate concentration was measured 5min after PBS and lactate injection. The data are presented as the mean±SD, and significant differences between the two groups were analyzed with the independent-samples *t*-test. ^*^ *p*<0.05 vs. 0min.

The lactate concentration of gastrocnemius following acute intramuscular administration of lactate is shown in Figure 1. The muscle lactate concentration after PBS injection is 2.86±0.22μmol/g muscle weight. Lactate level reached 9.7586±1.49μmol/g muscle weight at 5min after acute intramuscular lactate injection (*p*<0.05).

### The Time-Dependent Effects of Acute Intramuscular Lactate Injection on Blood Lactate Concentration

**Figure 2:**
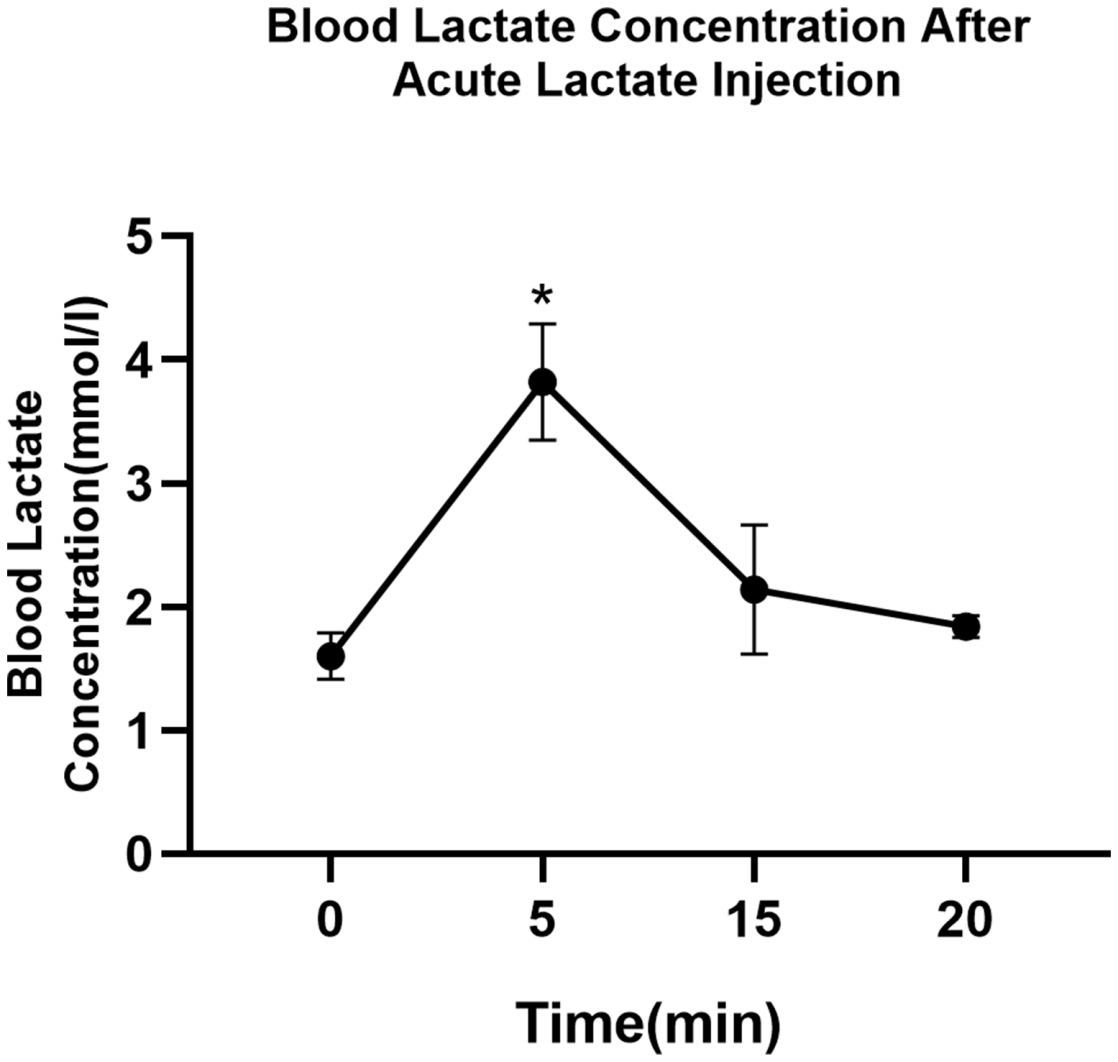
The time-dependent effects of intramuscular lactate-injection on blood lactate level. The blood lactate was determined at rest and 5, 10, 15, and 20min after the injection. The data are presented as the mean±SD, and significant differences between two groups were analyzed with the independent-samples *t*-test. ^*^ *p*<0.05 vs. 0min.

The blood lactate concentration in mice following acute intramuscular administration of lactate is shown in Figure 2. The average resting blood lactate concentration of mice is 1.6±0.1mmol/L. Lactate level significantly increased after acute intramuscular lactate injection and reached peak value (3.8±0.4mmol/L) at 5min (0 vs. 5min; *p*<0.05) and returned to baseline at 20min.

### The Time-Dependent Effects of Acute Lactate and Forskolin Injection on the cAMP-PKA Pathway

**Figure 3:**
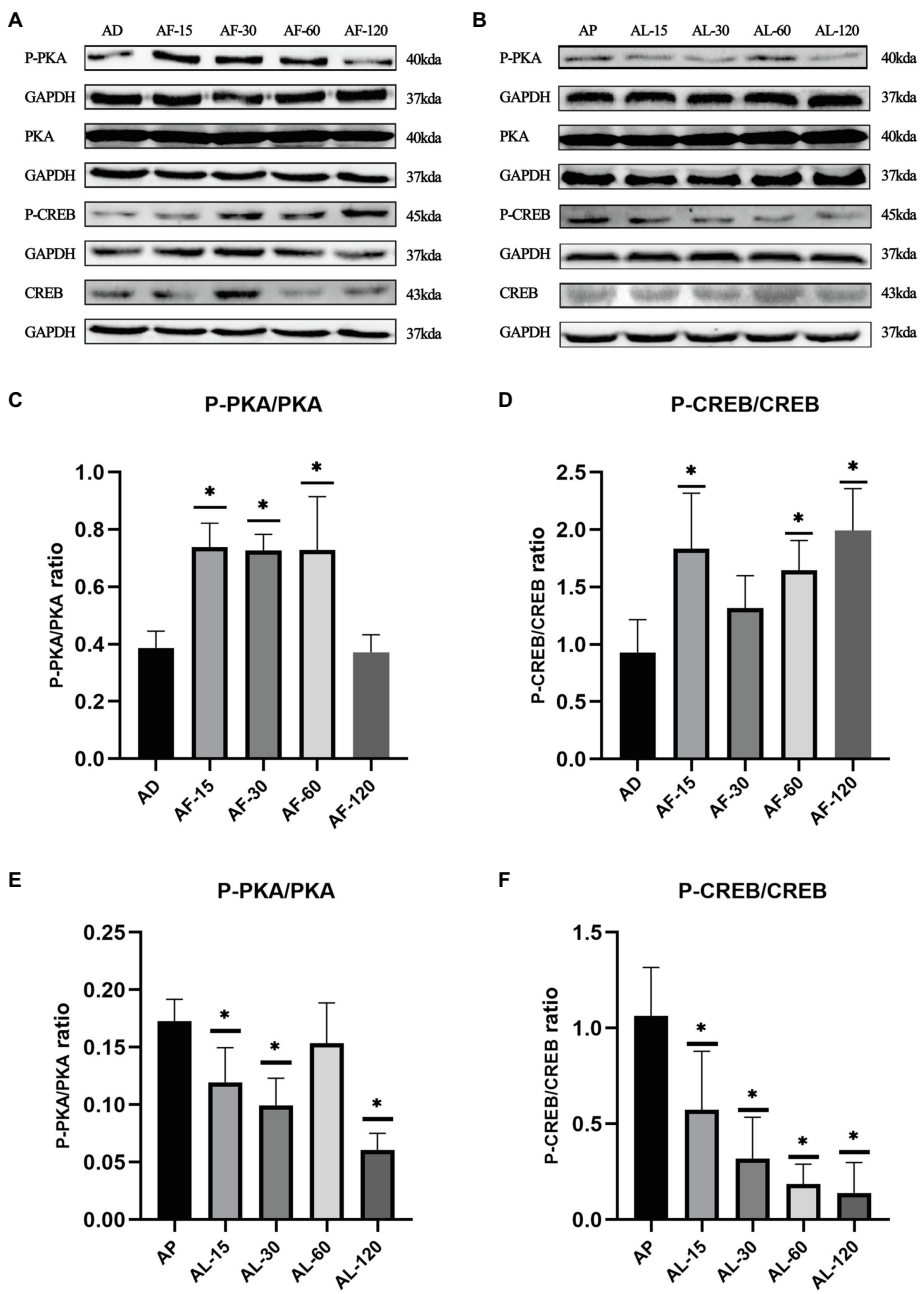
The time-dependent effects of acute lactate and forskolin injection on the cAMP-PKA pathway. **(A)** Western blot analysis of cAMP-PKA pathway proteins after acute forskolin injection. **(B)** Western blot analysis of cAMP-PKA pathway proteins after acute lactate injection. **(C,D)** The ratios of P-PKA/PKA and P-CREB/CREB after acute forskolin injection. **(E,F)** The ratios of P-PKA/PKA and P-CREB/CREB after acute lactate injection. AL-15, AL-30, AL-60, and AL-120 represent 15, 30, 60, and 120min after acute lactate injections. AF-15, AF-30, AF-60, and AF-120 represent 15, 30, 60, and 120min after acute forskolin injections. AP and AD mean sacrificed after acute PBS and DMSO injection, respectively. Three bands are used for statistics. The data are presented as the mean±SD, and significant differences between the two groups were analyzed with the independent-samples *t*-test. ^*^ *p*<0.05 vs. AD and AP.

As shown in Figure 3, the ratios of P-PKA/PKA (Thr198) and P-CREB/CREB (Ser133) in gastrocnemius decreased after acute lactate injection, while acute forskolin injection upregulated the ratios of P-PKA/PKA and P-CREB/CREB. Furthermore, the effects of acute lactate treatment were most obvious 30min after the injection, and the effects of acute forskolin treatment were most obvious 15min after the injection.

### The Blocking Effects of Acute Forskolin Treatment on Acute Lactate Injection Induced Inhibition of cAMP-PKA Pathway

**Figure 4:**
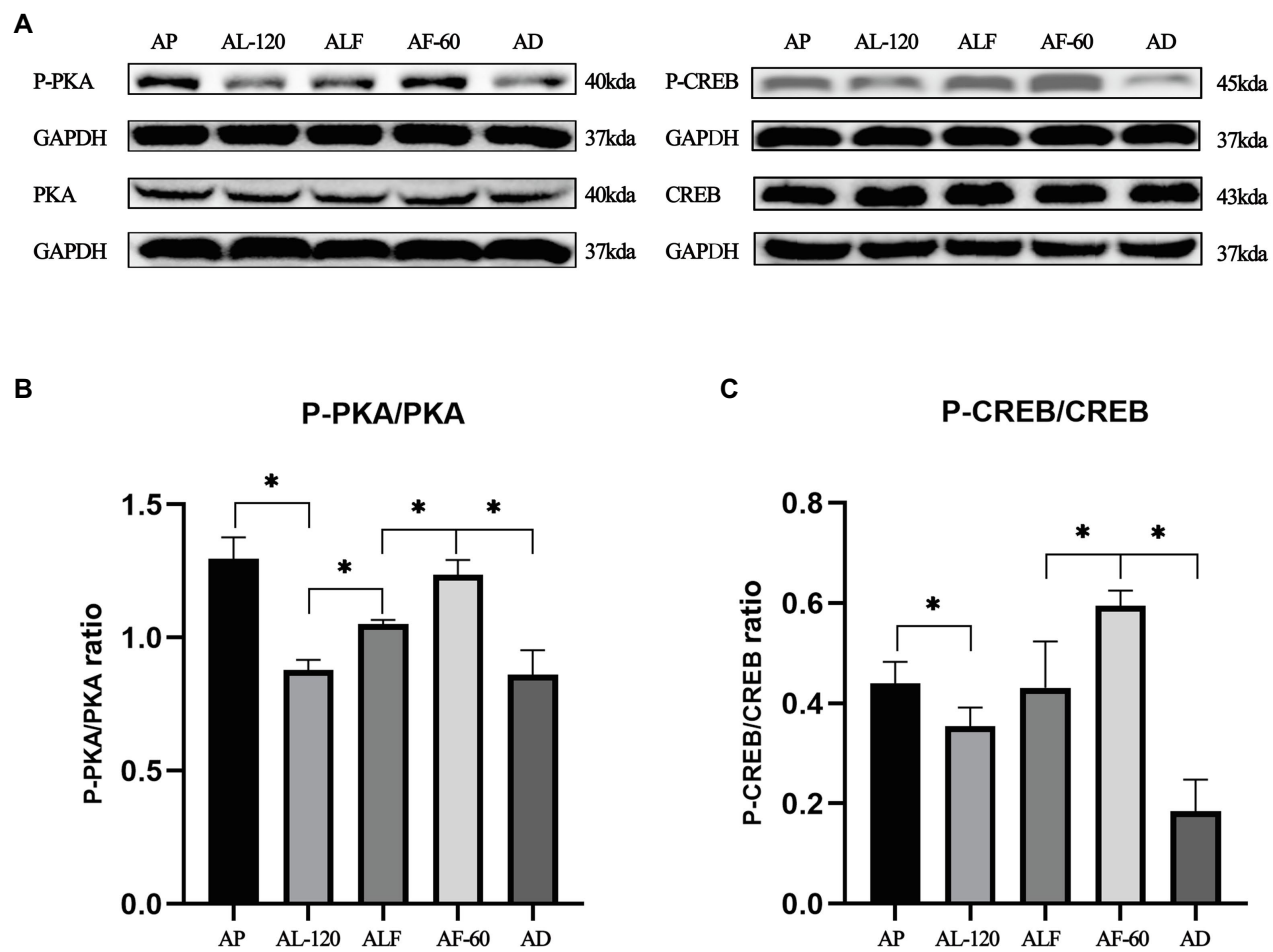
The blocking effects of acute forskolin treatment on acute lactate injection induced inhibition of cAMP-PKA pathway. **(A)** Western blot analysis of cAMP-PKA pathway proteins after acute lactate and forskolin injections. **(B)** The ratios of P-PKA/PKA after acute lactate and forskolin injection. **(C)** The ratios of P-CREB/CREB after acute lactate and forskolin injection. AP and AD mean sacrificed after acute PBS and DMSO injection, respectively. ALF means sacrificed at 60min after acute lactate and forskolin injection (60min interval). AL-120: sacrificed 120min after acute lactate injection. AF-60: sacrificed 60min after acute forskolin injection. Three bands are used for statistics. The data are presented as the mean±SD, and significant differences among the groups were analyzed with one-way ANOVA. ^*^ *p*<0.05.

As shown in Figure 4, we also validated forskolin’s blocking effect on lactate-induced inhibition of cAMP-PKA pathway. Indicated by the ratios of P-CREB/CREB and P-PKA/PKA in AL-120, ALF, and AF-60 group, the effects of acute lactate injection on cAMP-PKA pathway were blocked by acute forskolin injection (values of *p* less than 0.05).

### The Effects of Chronic Lactate and Forskolin Treatment on Intramuscular Triglyceride

**Figure 5:**
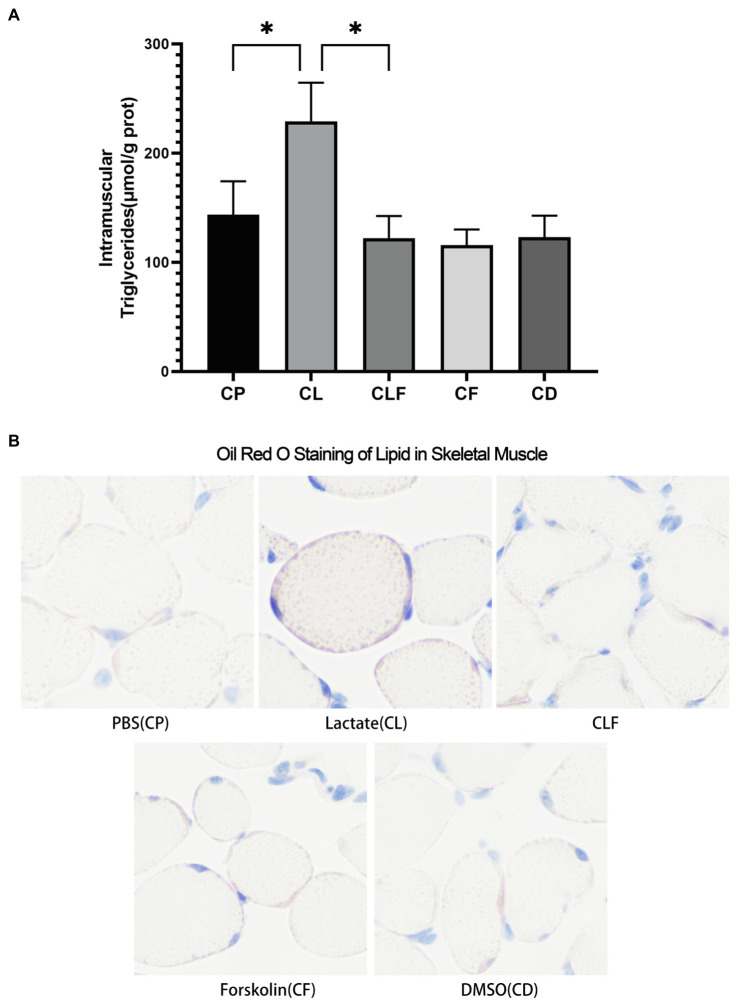
Intramuscular triglyceride variation after chronic administration. **(A)** Intramuscular Triglycerides Abundance of Skeletal Muscle (*n*=6). **(B)** The Oil Red O Staining of gastrocnemius of mice (*n*=5). CP, chronic PBS treated group; CL, chronic lactate treated group; *CF*, chronic forskolin treated group; CD, chronic DMSO treated group; and CLF, chronic lactate and forskolin treated group. Other images can be found in the additional materials. Measurement data were expressed by mean±SD, and significant differences among the groups were analyzed with one-way ANOVA. ^*^ *p*<0.05.

As shown in Figure 5A, chronic lactate intervention significantly increased the abundance of triglycerides in the gastrocnemius (*p*<0.05) compared to that of the chronic phosphate-buffered saline intervention (the solvent of lactate solution). Intramuscular triglycerides contents after chronic forskolin intervention were not downregulated compared to that of the chronic DMSO intervention (the solvent of forskolin solution). Moreover, the triglycerides accumulation effect of lactate was blocked by forskolin injection indicated by the results of the CLF group compared to that of the CL group (*p*<0.05). These changes coincide with the results of oil red staining (Figure 5B).

### The Effects of Chronic Lactate and Forskolin Treatment on Intramuscular GPR81 and the cAMp-PKA Pathway

**Figure 6:**
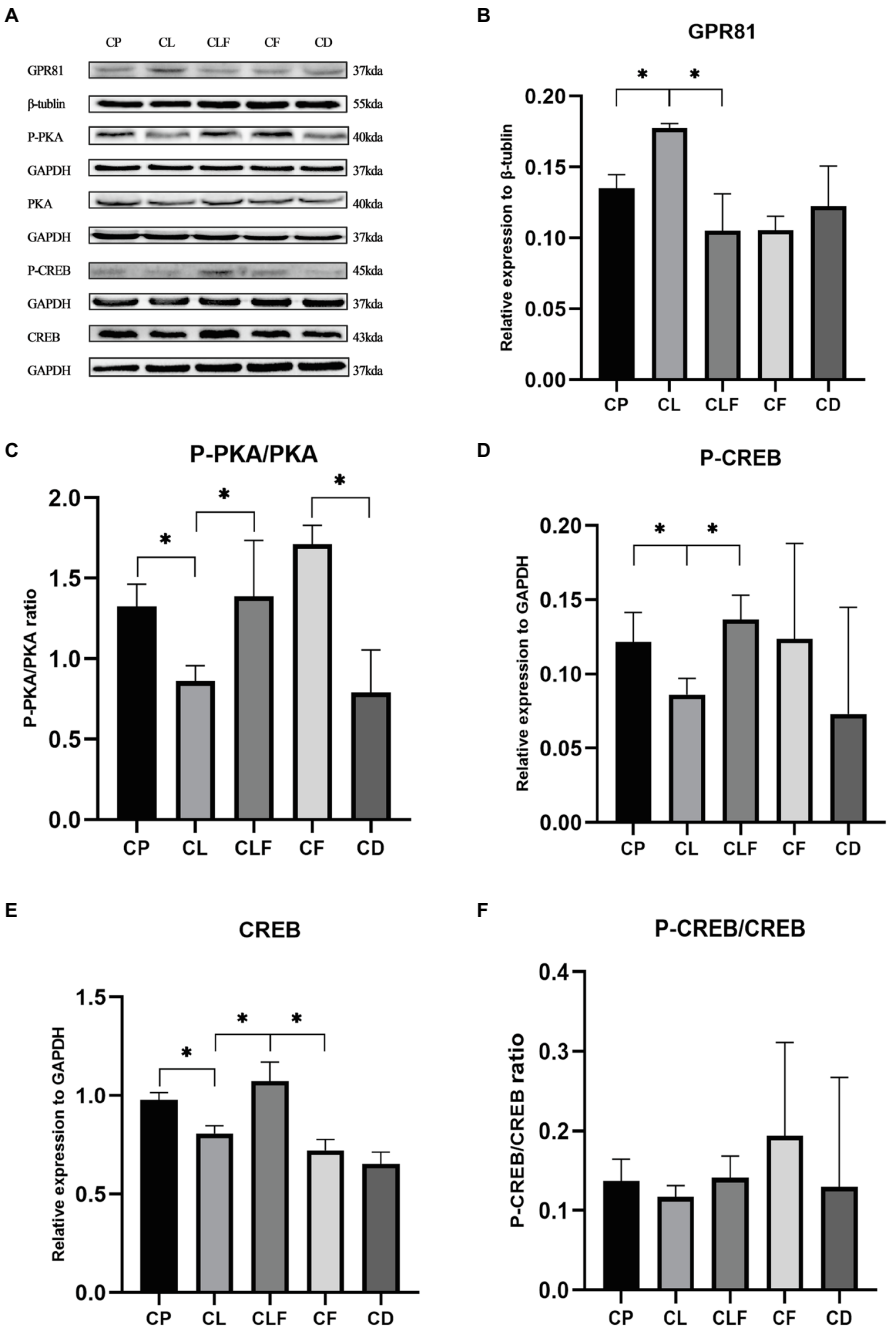
Western blot analysis and relative fold protein expression of GPR81, P-CREB, CREB, and the ratios of P-PKA/PKA and P-CREB/CREB after chronic administration. **(A)** Western blot analysis of GPR81 and proteins involved in cAMP-PKA pathway. **(B)** Fold protein expression of GPR81. **(C)** The ratio of P-PKA/PKA. **(D)** Fold protein expression of P-CREB. **(E)** Fold protein expression of CREB. **(F)** The ratio of P-CREB/CREB. Three bands are used for statistics. The data are presented as the mean±SD, and significant differences among the groups were analyzed with one-way ANOVA. ^*^ *p*<0.05.

As presented in Figure 6, chronic lactate treatment elevated the intramuscular expression of GPR81 in the CL group compared to that of the CP group (*p*<0.05). The ratios of P-PKA/PKA (Ser133), the expression level of CREB and the phosphorylation level of CREB (Thr198) in the CL group were decreased after chronic lactate injection compared with the CP group (*p*<0.05). Moreover, as indicated by the changing trend in the CLF group and CL group, the effects of lactate were blocked by forskolin injection (values of *p* less than 0.05). These results suggest that the chronic intramuscular injection of lactate successfully activated GPR81 to inhibit the cAMP-PKA pathway in skeletal muscle and forskolin is an effective inhibitor to block lactate’s function on the cAMP-PKA pathway.

### The Effects of Chronic Lactate and Forskolin Treatment on Intramuscular Lipid Metabolism-Related Proteins

**Figure 7:**
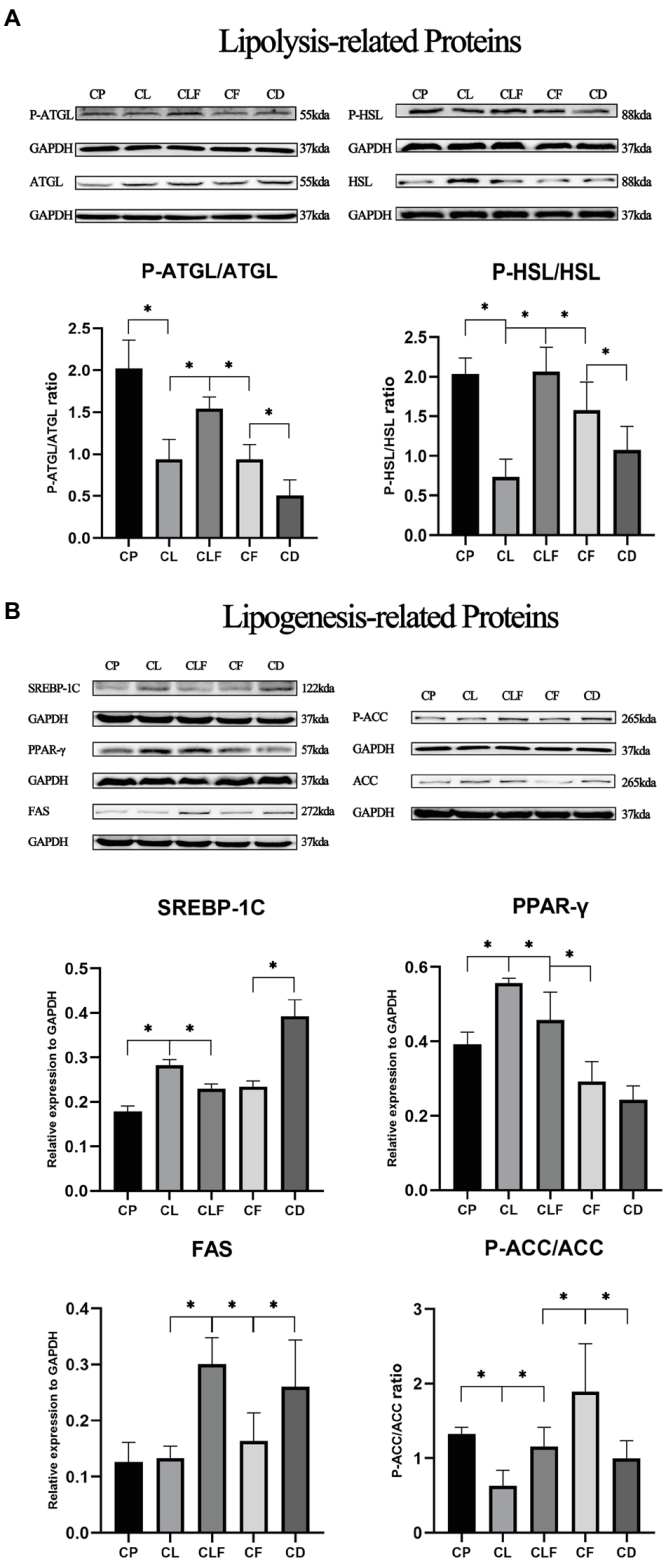
The expression levels of lipid metabolism-related proteins after chronic lactate and forskolin injection. **(A)** Western blot analysis and relative fold protein expression of lipolysis-related proteins. **(B)** Western blot analysis and relative fold protein expression of lipogenesis-related proteins. CP, chronic PBS treated group; CL, chronic lactate treated group; *CF*, chronic forskolin treated group; CD, chronic DMSO treated group; and CLF, chronic lactate and forskolin treated group. Relative expression levels were normalized to GAPDH. Three bands are used for statistics. The data are presented as the mean±SD, and significant differences among the groups were analyzed with one-way ANOVA. ^*^ *p*<0.05.

To further investigate the mechanism of lactate-induced intramuscular triglyceride variation, we measured the expression levels of lipogenesis-related and lipolysis-related proteins in gastrocnemius after chronic injections. Figure 7 provides that chronic lactate injection suppressed the phosphorylation of HSL (phospho S853), ATGL (phospho S406), and ACC (phospho S79), and upregulated the expression levels of SREBP and PPAR-γ (values of *p* less than 0.05). While chronic forskolin injection increased the phosphorylation levels of HSL, ATGL, and ACC, and downregulated the expression level of SREBP-1C (values of *p* less than 0.05) but not that of PPAR-γ. As indicated by the comparison between CLF group and CL group, lactate’s effects on these proteins were blocked by forskolin injection (values of *p* less than 0.05). Moreover, our results indicated that the expression of FAS was not modulated after chronic lactate injection.

### The Effects of Chronic Lactate and Forskolin Treatment on Intramuscular Mitochondria Content Biomarkers

**Figure 8:**
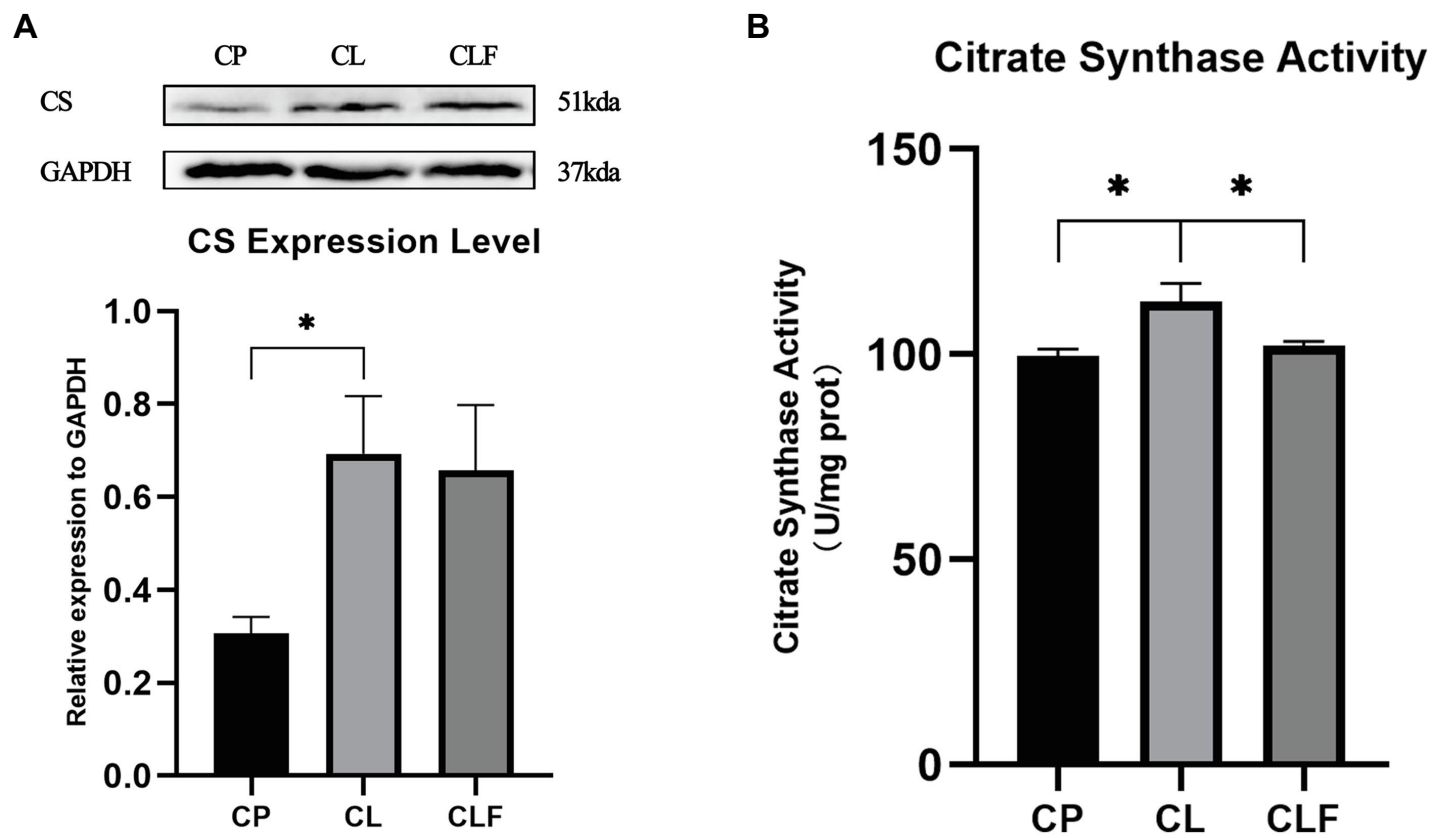
The activity and expression level of citrate synthase (CS). **(A)** The expression level of CS in gastrocnemius after chronic administration (*n*=8). **(B)** The activity of CS after chronic administration. CP, chronic PBS treated group; CL, chronic lactate treated group; and *CF*, chronic forskolin treated group. Relative expression levels were normalized to GAPDH. Three bands are used for statistics. The data are presented as the mean±SD, and significant differences among the groups were analyzed with one-way ANOVA. ^*^ *p*<0.05.

Exercise-induced intramuscular triglyceride accumulation is always accompanied by mitochondria adaption to hydrolyze triglycerides more efficiently. To investigate the impact of lactate on mitochondria and its mechanism, the biomarkers of mitochondria content: CS expression level and its activity were investigated. As illustrated in Figure 8, the expression level of CS and its activity increased after chronic lactate injection (*p*<0.05). Lactate’s effect on the activity of CS was blocked by forskolin (*p*<0.05). However, lactate-induced high expression of CS was not inhibited by the forskolin injection.

### The Effects of Acute Lactate Injection on the Contents of Lactic Acid and TCA Related Metabolites in Mitochondria

**Figure 9:**
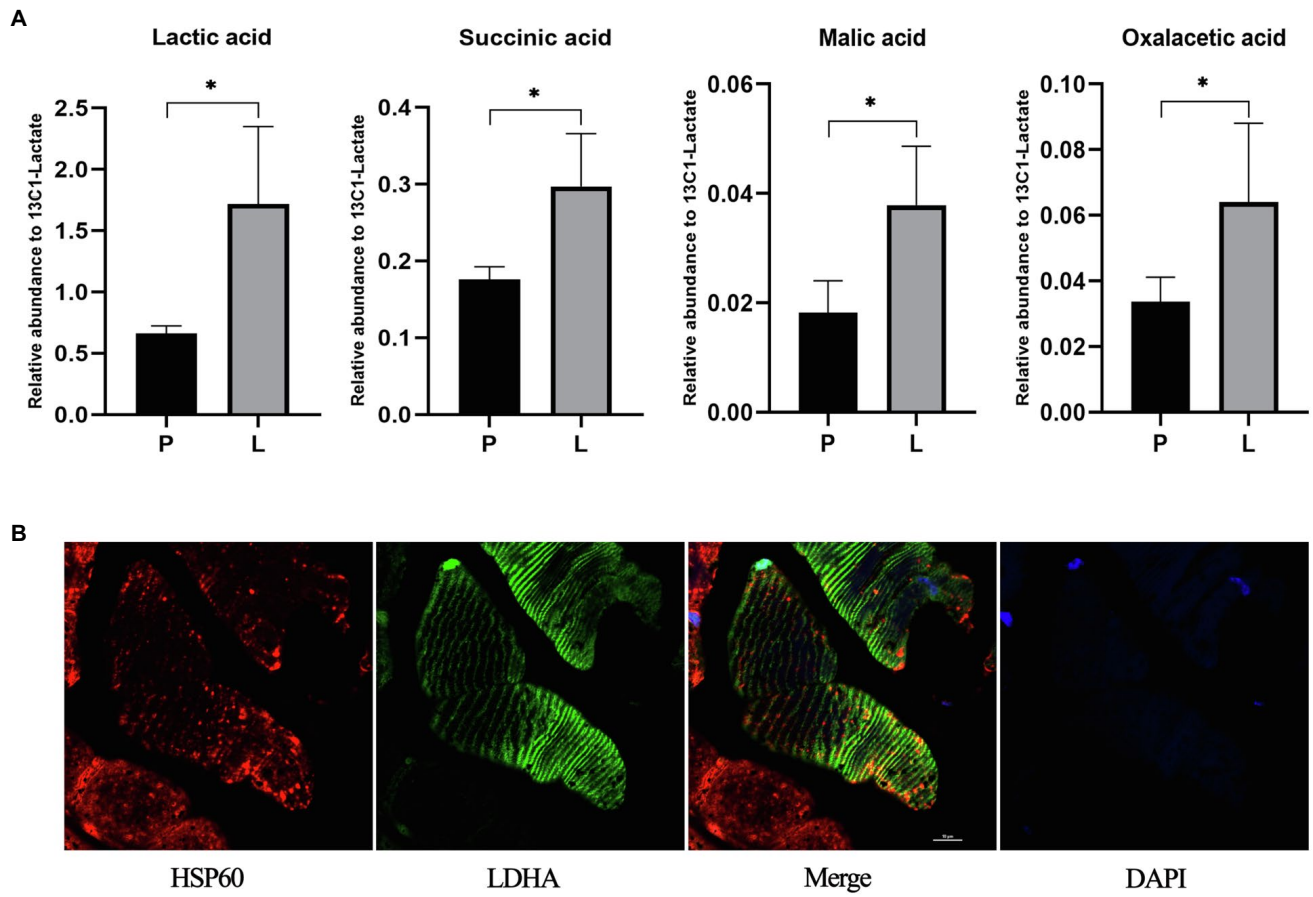
The contents of lactic acid and TCA related metabolites in mitochondria after acute lactate injection and the colocalization of mitochondria and LDHA. **(A)** The relative abundance of Lactic acid, Succinic acid, Malic acid, and Oxalacetic acid in mitochondria. P: after acute PBS injection (*n*=3); L: after acute lactate injection (*n*=3). The data are presented as the mean±SD, and significant differences between the two groups were analyzed with the independent-samples *t*-test. ^*^ *p*<0.05. **(B)** Confocal laser scanning microscopic imaging of immunolabeled LDH (green), and HSP60 (red). Antibodies against HSP60 were used as a label of the mitochondria.

The unexpected expression level of CS after chronic lactate treatment suggests other mechanisms might also contribute to the lactate-induced mitochondria content increase. Hence, we performed exploratory research on the potential metabolic mechanism of the lactate-induced mitochondria content increase. In the first place, to clarify whether injected lactate enters mitochondria and be oxidized there, we measured the contents of L-lactic acid and TCA-related metabolites in mitochondria after acute lactate injection. As shown in Figure 9A, the contents of L-lactic acid, succinic acid, L-malic acid, and oxalacetic acid after lactate injection were significantly increased compared to PBS injection (the value of *p* of each analysis is less than 0.05).

## Colocalization of Mitochondria and LDHA

After clarified that injected lactate could be transported into mitochondria and oxidized there, we further validated the prerequisite for lactate oxidation in mitochondria. As shown in Figure 9B, LDHA exists in mitochondria of the gastrocnemius muscle, which provides a possibility for lactate oxidation in mitochondria and metabolism-related mechanisms of lactate-induced mitochondria content increase.

## Discussion

We have previously reported that lactate contributed to intramuscular triglyceride accumulation and mitochondria adaption in rats. However, the cellular/molecular mechanisms were unclear. The role of cAMP-PKA pathway in lactate-induced lipid accumulation in adipose tissue has been widely reported in previous studies (Sakurai et al., 2014). Moreover, CREB (downstream of cAMP-PKA pathway) is also reported to be associated with mitochondria biogenesis and content increase.

In the present study, we identified, for the first time, that cAMP-PKA pathway is involved in lactate-induced intramuscular triglyceride accumulation. We also explored possible mechanisms of increased mitochondria content after chronic lactate injection. The unique results ascertained in these trials were as follows: (1) chronic intramuscular lactate injection promoted lipid accumulation by suppressing lipolysis and stimulating lipogenesis. The suppressed lipolysis and stimulated lipogenesis were partially blocked by forskolin; (2) CS content and its activity increased after chronic intramuscular lactate injection, and the lactate-induced CS content change was not blocked by forskolin treatment; and (3) the contents of lactate-related metabolites in skeletal muscle mitochondria increased after acute lactate injection, and LHDA was also validated to exist in mitochondria. These results provide a possibility for metabolism-related mechanisms of lactate-induced mitochondria content increase.

High-intensity training is widely known to induce intramuscular lipid accumulation (van Loon and Goodpaster, 2006; Shaw et al., 2012). The adaptation of intramuscular triglyceride storage after exercise may benefit exercising tissue by supplying free fatty acids (Zacharewicz et al., 2018). However, the molecular mechanism underlying this adaptation is unknown. Our prior studies have provided evidence that lactate might be one of the triggers (Zhou et al., 2021b). In that study, we have identified that after one-time high-intensity exercise, the average blood lactate concentration in rats was 5.08±1.38mmol/L. According to previous studies, using a certain dose [0.64ml/kg lactate (0.25M)] of lactic acid solution injected into the gastrocnemius muscle of rodents can simulate exercise-induced lactate changes (Nikooie and Samaneh, 2016; Zhou et al., 2021b). Here, we tested the previously established injection protocol in mice and found that muscle lactate concentration reached 9.7586±1.49μmol/g muscle weight and the blood lactate level reached 3.8±0.4mmol/L after the injection, which is close to exercise-induced changes in blood lactate. Therefore, we used 0.25M lactate solution in a dose of 0.64ml/kg to mice’s gastrocnemius to stimulate the transient elevation of systemic lactate levels after exercise.

The role of GPR81 and cAMP-PKA pathway in lactate-induced lipid accumulation has been widely reported in previous studies on adipose tissue. For example, lactate-induced suppression of lipolysis in explants of white adipose tissue (WAT) has been confirmed to depend on the presence of GPR81 (Sakurai et al., 2014), which is closely related to the inhibition of cAMP-PKA pathway (Brown et al., 2020). It is also illustrated that lactate activates the G protein-coupled receptor GPR81 in adipocytes and mediates antilipolytic effects through Gi-dependent inhibition of adenylyl cyclase (Ahmed et al., 2010). In contrast to lactate, forskolin has been long recognized as a classic activator of the cAMP-PKA pathway (Sakurai et al., 2014). Hence, in this study, forskolin was selected as the inhibitor of lactate to investigate whether lactate’s effects on intramuscular triglyceride accumulation and mitochondria content are modulated by the cAMP-PKA pathway.

**Figure 10:**
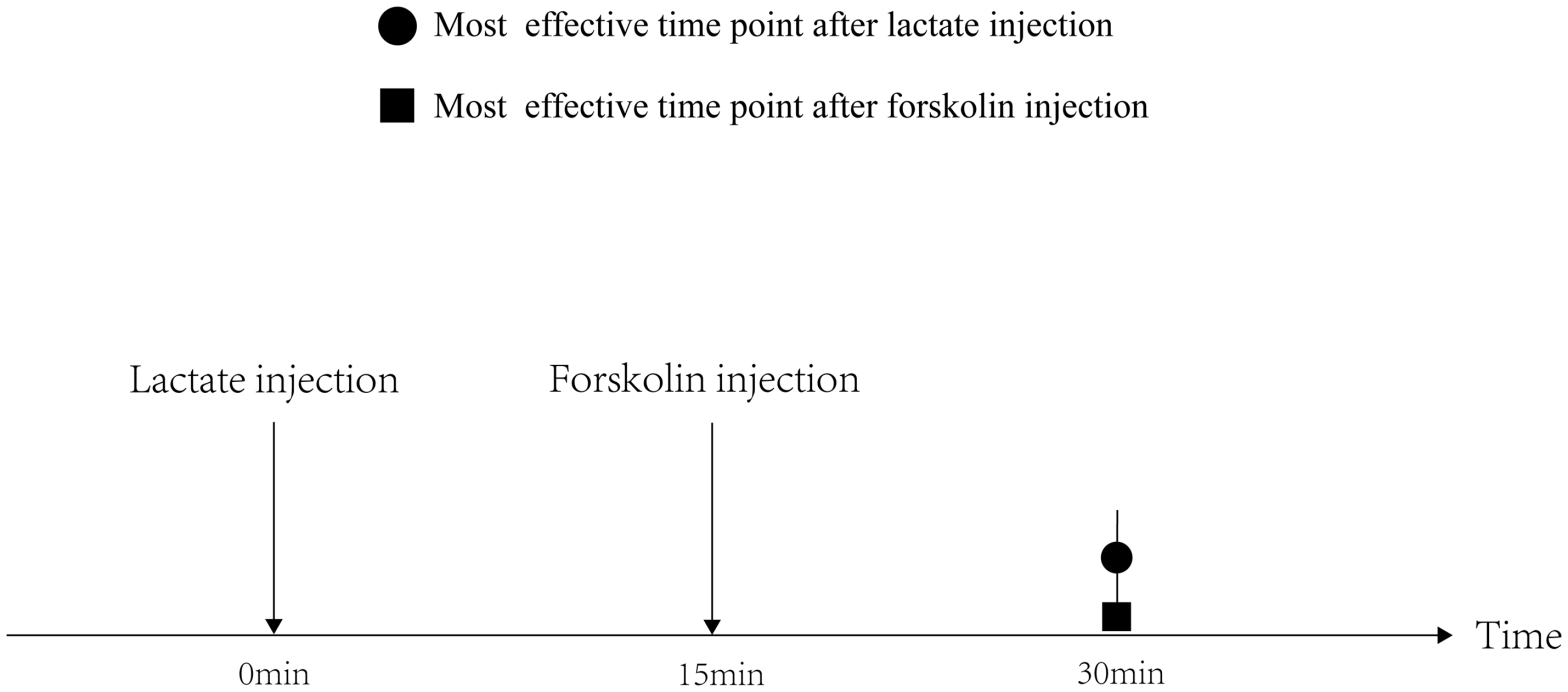
Effectivity of lactate and forskolin injection on cAMP-PKA pathway.

Before the chronic administration, we explicated the time-dependent effects of acute lactate and forskolin injections on the intramuscular cAMP-PKA pathway and the forskolin’s inhibitory effects on lactate. Results showed that the ratios of P-PKA/PKA and P-CREB/CREB increased after acute forskolin injection, while acute lactate injection suppressed the expression of these proteins. Forskolin’s inhibitory effect on lactate was validated by the expression levels of ALF group. Furthermore, lactate’s inhibitory effects on the cAMP-PKA pathway were most obvious at 30min after injection. Forskolin’s activation effects on the cAMP-PKA pathway were most obvious at 15min after the injection. Hence, we selected an interval of 15min between lactate injection and forskolin injection in chronic administration to ensure the forskolin’s inhibitory effects on lactate’s function (Figure 10).

Lactate’s role on lipid accumulation in adipocytes has been demonstrated (Gold et al., 1963; Bjorntorp, 1965; Houghton et al., 1971; Boyd et al., 1974; De Pergola et al., 1989; Cai et al., 2008), and the mechanism of it might be lactate activates its receptor GPR81 and then inhibit the cAMP-PKA pathway to inhibit lipolysis (Sakurai et al., 2014). Whether lactate results in a similar process in skeletal muscle is unclear. In this study, the abundance of triglyceride in the gastrocnemius increased significantly after lactate intervention compared to that of the chronic phosphate-buffered saline intervention. This effect of lactate was significantly blocked by forskolin injection, suggesting lactate-induced intramuscular triglyceride accumulation is regulated by the cAMP-PKA pathway. The results of the ratios of P-PKA/PKA and P-CREB/CREB also confirmed this point of view. However, one unanticipated finding was that there was no significant difference between the intramuscular triglyceride contents in the chronic DMSO treatment group and the forskolin treatment group. This result may be explained by the hypothesis that a self-protection mechanism might be activated to prevent fatty acids from being hydrolyzed (Zhou et al., 2021a) when the intramuscular abundance of triglycerides was not elevated by external intervention, and further investigation is suggested to validate it.

Previous studies have found that lactate-induced intramuscular triglycerides accumulation coincides with the inhibited lipolysis and promoted lipogenesis (Zhou et al., 2021b). Here, to further investigate the role of the cAMP-PKA pathway in lactate-induced intramuscular triglyceride accumulation, we measured the expression levels of lipolysis-related proteins (P-HSL/HSL and P-ATGL/ATGL) and lipogenesis-related proteins (SREBP, PPAR-γ, FAS, and P-ACC/ACC) in gastrocnemius after chronic administration. ATGL plays a key role in lipid droplet/adiposome degradation (Smirnova et al., 2006). ATGL catalyzes the initial step in triglyceride hydrolysis in adipocytes, and it is also reported to regulate triglyceride hydrolysis in muscle recently (Smirnova et al., 2006; Zhao et al., 2020). HSL is believed to play a regulatory role in initiating the degradation of intramuscular triacylglycerol in skeletal muscle (Watt and Spriet, 2004). It is known to be the rate-limiting enzyme for hydrolysis of triacylglycerol in adipocytes at first, and also be proved to regulate the intramyocellular hydrolysis of triacylglycerol to diacylglycerol and further on to monoacylglycerol (Donsmark et al., 2005). In this study, the phosphorylation of HSL and ATGL was suppressed after chronic lactate injection, and this variation was successfully blocked by forskolin injection. However, the phosphorylation levels of HSL and ATGL in CLF group were higher than those in the *CF* group. A possible explanation for this might be that repetitive injection stimulation induced chronic stress and led to the activation of HSL and ATGL. Further study is needed to validate this hypothesis since previous study only investigated the expression of mRNA in HSL and ATGL under chronic stress (Mu et al., 2019). Meanwhile, it is also indicated that other mechanisms might contribute to the lactate-induced intramuscular triglyceride accumulation. Hence, our results of lipolysis-related proteins suggest, to a great extent, the lactate-induced intramuscular triglyceride accumulation is achieved by inhibition of lipolysis, and this process is regulated by the cAMP-PKA pathway.

Correspondingly, lipogenesis-related proteins were also investigated in this study. SREBP-1C is a bound transcription factor that activates genes encoding enzymes required for the synthesis of cholesterol and unsaturated fatty acids (Debose-Boyd and Ye, 2018). The cAMP/PKA pathway is recognized to negatively regulate SREBP-1C expression at the transcriptional and post-translational levels (Zhou et al., 2007). Here, the expression of SREBP-1C was promoted after chronic lactate injection and this variation was inhibited by forskolin injection, though no difference was observed between the SREBP expression levels in the CLF group and *CF* group. Therefore, cAMP-PKA-SREBP-1C pathway might contribute to lactate-induced intramuscular triglyceride accumulation, and other mechanisms besides this pathway might also play a role. PPAR-γ is also an important lipogenesis-related protein (Choi et al., 2011; Koves et al., 2013). According to our results, PPAR-γ might contribute to lactate-induced intramuscular triglyceride accumulation and this process might be regulated by the cAMP-PKA pathway, but no significant difference between CD and *CF* group suggests other mechanisms might also regulate PPAR-γ. FAS is crucial for the synthesis of saturated long-chain fatty acids and it is also reported to be regulated by SREBP-1C (Semenkovich, 1997; Griffin et al., 2007; Roder et al., 2007; Choi et al., 2008). However, no significant difference was observed after lactate injection and the expression levels of FAS in CLF, *CF*, and CD groups are confusing. This result may be explained by the fact that FAS is also regulated by many hormones (Donkin et al., 1996; Najjar et al., 2005; Xu et al., 2005), and further study is needed to explicate it. ACC catalyzes the formation of malonyl-CoA, an essential substrate for fatty acid synthesis in lipogenic tissues and a key regulatory molecule in muscle, brain, and other tissues (Wakil, 1958; Brownsey et al., 2006). The phosphorylation of ACC changes the ACC from the active form of a large linear polymer to the inactive form (Cho et al., 2010), therefore, the low phosphorylation level of ACC represents promoted lipogenesis (Underwood et al., 2007; Labrie et al., 2015; Sun et al., 2019). In this study, the phosphorylation of ACC was downregulated after chronic lactate injection and this variation was blocked by forskolin injection. The above results of lipogenesis-related proteins imply that the lactate-induced intramuscular triglyceride accumulation might be achieved by promoting the expression of lipogenesis-related proteins, and mechanisms besides cAMP-PKA pathway might also play a prominent role in this process.

Previous studies have validated that intramuscular triglyceride accumulation is accompanied by mitochondria biogenesis and content increase in skeletal muscle (Koves et al., 2013). Our recent research also verified that lactate injection could increase the expression levels of mitochondria content biomarkers, but the mechanism remains unknown. Here, we implemented a tentative research to explore the metabolism-related possibility of the intramuscular mitochondria content increase after lactate injection. CS content and its activity have long been recognized as biomarkers of mitochondrial content (Nederlof et al., 2017; Gil et al., 2019; Wang et al., 2020). According to our results, the activity of CS was promoted after chronic lactate injection and this variation was inhibited by forskolin injection. However, the changes in CS activity were of low magnitude (CL is 13.3% higher than CP, and CLF is 9.5% lower than CL). The expression level of CS was also elevated after lactate injection (*p*<0.05), and this effect was not inhibited by forskolin injection as indicated by the expression levels of the CL and CLF groups. This suggests the expression of CS might not be regulated by the cAMP-PKA signaling pathway, and other mechanisms (e.g., metabolic mechanism) of the lactate-induced mitochondria content increase might exist.

Hence, we performed explorative research for the potential mechanisms. We found that the contents of lactate-related metabolites in the mitochondria of skeletal muscle increased after acute lactate injection, and LHDA was also validated to exist in mitochondria in this study. These results provide a possibility for metabolism-related mechanisms of lactate-induced mitochondria content increase: lactate could enter mitochondria and be oxidized there, which might induce an adaptive increase of mitochondria content.

CREB has long been recognized as a nuclear transcription factor (Yan et al., 2016). In addition to its functions in the nucleus, it is also imported into the mitochondria and activates transcription of mitochondria proteins (Lee et al., 2005; Wu et al., 2006; Bergman and Ben-Shachar, 2016). In this study, lactate injection decreased phosphorylation of CREB, which might suppress mitochondrial transcription. This seemingly contradictory phenomenon might actually indicate the presence of the metabolism-related mechanisms, and the metabolism-induced mitochondria adaption might be stronger than the effects of CREB.

Composed of mMCT1, CD147 (basigin), mLDH, and cytochrome oxidase (COX), mLOC plays a key role in the oxidation of lactate in mitochondria (Hashimoto and Brooks, 2008). MCT4 is also always mentioned together with MCT1 as important lactate transporters in muscle (Bonen, 2001). Hence, mLOC could be a unique complex that warrants further attention to test our hypothesis.

In addition to metabolism-related mechanisms, the possibility of other potential mechanisms of lactate-induced mitochondrial biogenesis might exist. It has been reported that lactate could stimulate ROS generation, which activates transcription factors including not only CREB, but also factor-kappaB, nuclear factor erythroid-2, and nuclear respiratory factor-2 (Hashimoto and Brooks, 2008). Hence, transcription factors besides CREB might also contribute to lactate-induced mitochondria biogenesis and content increase, this seems to be a possible explanation for the results that forskolin (CREB activator) failed to fully suppress lactate-induced mitochondria biogenesis and content. In this study, the activity and expression level of CREB was inhibited, indicating the effects of GPR81-cAMP signaling pathway might be stronger than that of ROS. However, with very little evidence, this is only a hypothesis that needs to be confirmed by future studies.

In conclusion, based on our findings from previous and current studies, we propose that the lactate-induced intramuscular triglyceride accumulation is achieved by inhibition of lipolysis, and this process is regulated by the cAMP-PKA pathway. Suppressed lipogenesis also contributes to the lactate-induced triglyceride accumulation, and this process might be regulated by the cAMP-PKA pathway. Lactate injection might increase mitochondria content and cAMP-PKA pathway might have a limited contribution to it, while lactate-related metabolism process and various ROS-related transcription factors might play a role.

## Limitation

Relative to the total volume of a mouse gastrocnemius, the volume of injection into the gastrocnemius was high in this study. This might mechanically disrupt the muscle bed and influence the experimental results. Additionally, this study only implemented a tentative research to explore the metabolism-related possibility of the intramuscular mitochondria content increase after lactate injection. Future study is needed to validate our hypothesis.

## Data Availability Statement

The original contributions presented in the study are publicly available. This data can be found at: https://figshare.com/s/da4fa6da5efaccbcb7ad.

## Ethics Statement

The animal study was reviewed and approved by Medical Ethics Committee of Sichuan University.

## Author Contributions

All authors listed have made a substantial, direct and intellectual contribution to the work, and approved it for publication.

## Funding

This project was funded by Fundamental Research Funds for the Central Universities of China and the National Natural Science Foundation of China (31801002).

## Conflict of Interest

The authors declare that the research was conducted in the absence of any commercial or financial relationships that could be construed as a potential conflict of interest.

## Publisher’s Note

All claims expressed in this article are solely those of the authors and do not necessarily represent those of their affiliated organizations, or those of the publisher, the editors and the reviewers. Any product that may be evaluated in this article, or claim that may be made by its manufacturer, is not guaranteed or endorsed by the publisher.
